# Efficacy and safety of Osimertinib and other third-generation EGFR TKIs in advanced NSCLC: a systematic review and meta-analysis

**DOI:** 10.1007/s12672-025-04353-7

**Published:** 2026-01-07

**Authors:** Juan Lin, Chunxia Zhang, Jinshan Weng, Shanzuan Wang

**Affiliations:** 1 Respiratory and Critical Care Medicine, The Affiliated Hospital of Putian University, 999 Dongzhen East Road, Licheng District, Putian, 351100 China; 2 Departments of Clinical Medicine, Fujian Medical University, Fuzhou, P.R. China; 3 Departments of Geriatrics, The Affiliated Hospital of Putian University, 999 Dongzhen East Road, Licheng District, Putian, 351100 China

**Keywords:** Non-small cell lung cancer, Osimertinib, Epidermal growth factor receptor inhibitor, Clinical efficacy, Safety analysis

## Abstract

**Objective:**

To systematically evaluate the clinical efficacy, safety, and prognostic impact of third-generation epidermal growth factor receptor (EGFR) tyrosine kinase inhibitors (TKIs), particularly osimertinib, in patients with advanced non-small cell lung cancer (NSCLC).

**Method:**

A comprehensive search of international and Chinese databases was conducted from inception to April 2025 to identify randomized controlled trials (RCTs) assessing osimertinib in advanced NSCLC. Data extraction and quality assessment were performed independently by two reviewers using the Cochrane Handbook (version 5.3). Meta-analysis was conducted using RevMan 5.4.

**Result:**

Nine RCTs involving 1,880 patients were included. Osimertinib significantly improved objective response rate (ORR) [OR = 2.99, 95% CI: 1.87–4.77] and disease control rate (DCR) [OR = 2.80, 95% CI: 1.38–5.67] compared to control therapies. The incidence of grade ≥ 3 adverse events was significantly lower in the osimertinib group [OR = 0.26, 95% CI: 0.14–0.47]. Osimertinib increased the risk of dermatologic events such as paronychia and rash but decreased gastrointestinal symptoms including nausea and vomiting.

**Conclusion:**

Osimertinib is an effective and generally well-tolerated treatment option for patients with advanced NSCLC. While efficacy and safety are favorable, further research is needed to assess long-term outcomes and monitor serious adverse events.

## Introduction

Lung cancer is a highly fatal malignancy originating from the bronchial mucosa or alveolar epithelium. About 80% of lung cancer patients are non-small cell lung cancer (NSCLC), which has a 10% to 12% 5-year survival rate. Although chemotherapy remains a common treatment modality for advanced NSCLC, its effectiveness is often suboptimal. In recent years, advances in precision medicine have shifted therapeutic strategies toward targeted interventions. Due to their high specificity and efficacy, epidermal growth factor receptor tyrosine kinase inhibitors (EGFR-TKIs) have been adopted as the primary first-line therapy in advanced NSCLC individuals with epidermal growth factor receptor (EGFR) gene alterations. Although its initial response rate is relatively high, 20% to 30% of NSCLC individuals with EGFR gene mutations still develop primary drug resistance, and patients who respond effectively at the beginning of treatment may also develop acquired drug resistance during the treatment process. Among them, 50% to 60% of the drug resistance mechanisms are caused by T790M mutations [1, 2].

Osimertinib, a third-generation EGFR-TKI, demonstrates strong clinical effectiveness in individuals after progression on first- or second-generation EGFR-TKIs, with advanced NSCLC exhibiting the EGFR-T790M resistance mutation, while maintaining a relatively favorable safety profile [3]. In 2017, the China Food and Drug Administration gave it approval to treat advanced NSCLC. Clinical reports indicate that the objective response rate (ORR) and progression-free survival (PFS) of Osimertinib in the handling of advanced NSCLC with positive T790M mutation reached 61% and 9.6 months, respectively [4]. Meanwhile, because Osimertinib has a good blood-brain barrier penetration ability and can be used to treat central nervous system (CNS) metastases, it has become a commonly used clinical drug for the treatment of NSCLC [5].

The common Adverse drug reactions (ADRs) of Osimertinib in clinical practice include rash, diarrhea, dry skin, paronychia, etc. With the continuous deepening of research, some serious ADRs have gradually been discovered: cardiotoxicity [6, 7], pulmonary embolism, interstitial pneumonia [8, 9], severe liver damage [10], etc. Severe adverse reactions can lead to poor medication compliance in patients, reduced quality of life, and affect the rate of tumor progression. In the last several years, Several research have been conducted on osimertinib in the handling of NSCLC. This research seeks to assess the efficacy and safety of Osimertinib in the handling of advanced NSCLC with EGFR sensitive mutations by using the method of meta-analysis and screening and analyzing relevant literature, providing relevant basis for rational clinical drug use.

## Methods

### Literature materials

A comprehensive literature search was conducted across PubMed, EMBASE, ScienceDirect, the Cochrane Library, and major Chinese databases including CNKI, VIP, Wanfang, and CBM. Additional eligible studies were identified by manually screening the reference lists of included articles. The search aimed to identify randomized controlled trials (RCTs) evaluating the safety, efficacy, and prognostic impact of third-generation EGFR tyrosine kinase inhibitors, with a primary focus on osimertinib, in patients with advanced non-small cell lung cancer (NSCLC). Both Medical Subject Headings (MeSH) and free-text terms were used in the search strategy, including the keywords: “osimertinib,” “EGFR inhibitor,” “NSCLC,” “clinical efficacy,” and “safety analysis.” The search included studies published from January 2010 through April 2025. No language restrictions were applied. Gray literature was excluded. Two independent reviewers conducted the literature screening and study selection, and discrepancies were resolved through discussion or consultation with a third reviewer.

### Criteria for literature inclusion and exclusion

#### Inclusion criteria of literature

1. Study design: Phase II or III randomized controlled trials (RCTs).
2. Participants: Adult patients (aged ≥ 18 years) with histologically or cytologically confirmed advanced or metastatic non-small cell lung cancer (NSCLC) harboring EGFR mutations, as determined by genetic testing.
3. Interventions: The experimental group (EG) received osimertinib monotherapy, while the control group (CG) was treated with either platinum-based chemotherapy or other comparator regimens.
4. Outcomes: Efficacy: Objective response rate (ORR), disease control rate (DCR); Safety: Incidence of grade ≥ 3 adverse drug reactions (ADRs) and specific ADR types; Prognosis: Progression-free survival (PFS).

#### Exclusion criteria

Studies were excluded if they met one or more of the following conditions:

1. Non-randomized trials, including observational studies or single-arm trials without a control group.
2. Incomplete or missing outcome data that could not be extracted or obtained after contacting the study authors.
3. Duplicate reports—only the most comprehensive or latest version was included.
4. Studies with undefined or ambiguous primary outcome measures.
5. Review articles, conference abstracts, editorials, or meta-analyses.
6. Case reports or case series without a clearly defined comparator group.

### Data extraction and quality assessment

(1) Risk of bias assessment: The included studies’ risk of bias was evaluated using the “Risk of Bias” tool (version 5.3) from the Cochrane Handbook for Systematic Reviews of Interventions. This assessment addressed blinding, allocation concealment, sequence construction, selective reporting, inadequate outcome data, and other potential causes of bias. (2) Data extraction and literature screening: Two impartial reviewers evaluated the quality of the studies, retrieved pertinent data, and reviewed the literature. Any disputes were settled by dialogue or, if required, by speaking with a third reviewer. Literature management and reference tracking were conducted using NoteExpress software, while data extraction and organization were performed using Microsoft Excel. In order to get the information needed for studies with missing or insufficient data, efforts were undertaken to get in touch with the associated authors. Extracted data included the following:

(1) Fundamental research characteristics, such as sample size, year of publication, and initial author; (2) Intervention details – osimertinib monotherapy in the EG versus conventional chemotherapy or alternative treatments in the CG;

(3) Outcome measure: ORR, DCR, incidence of grade ≥ 3 ADRs, incidence of specific ADR types, and PFS.

### Statistical processing

Review Manager was used to do meta-analyses (RevMan, version 5.4)., provided by the Cochrane Collaboration. For constant variables such as PFS, 95% confidence intervals and weighted mean differences (WMDs) were computed. Inter-study heterogeneity (HET) was evaluated through Chi-square tests (χ²) and quantified using the I² statistic. *P* > 0.1 and I^2^ < 50% were considered minimum HET, and a fixed-effects model was used. Conversely,, a REM was used when *P* < 0.1 and I² ≥ 50%, indicating significant HET. When P-values were below 0.05 and the HET source remained unclear, meta-analysis was avoided, and a narrative synthesis was provided. The Egger’s test and funnel plots were designed to evaluate possible publication bias. If Egger’s test yielded a p-value less than 0.1, the Trim and Fill approach would be considered. However, considering that there were only ten research included, funnel plots were not constructed.

##  Results

### Literature screening results and baseline information of eligible studies

**Fig. 1:**
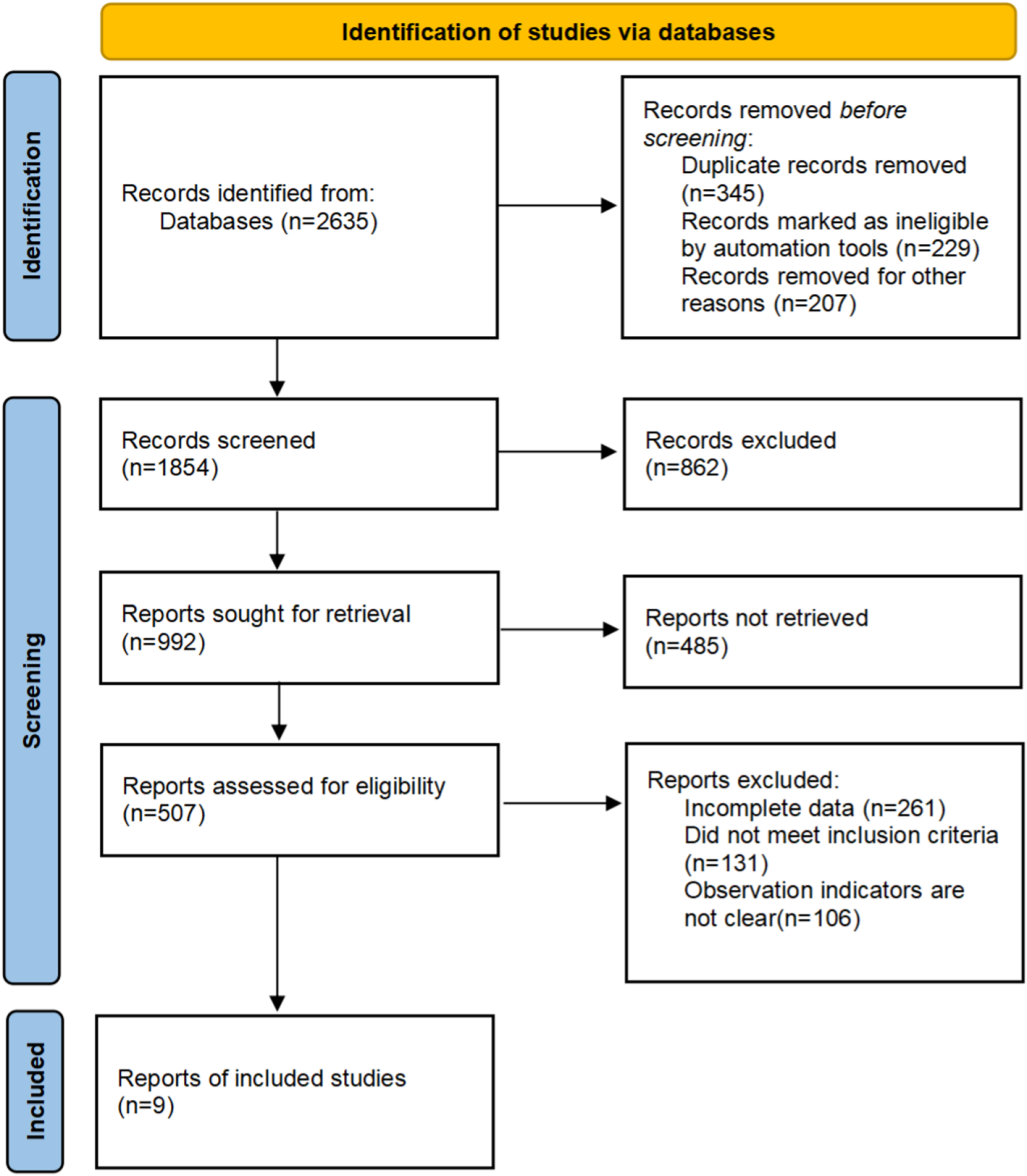
Diagram of the Study Selection Process

The process of literature identification and selection was carried out in compliance with the PRISMA (Preferred Reporting Items for Systematic Reviews and Meta-Analyses) guidelines. 2,635 items from the original pool were obtained through searches of electronic databases. After taking out duplicate entries, 1,854 unique studies remained. Preliminary screening of titles and abstracts yielded 992 potentially relevant articles. Following the exclusion of irrelevant studies, reviews, case reports, and non-randomized trials, the eligibility of 507 full-text publications was evaluated. Of these, 498 were excluded due to the absence of clearly defined primary outcome measures. In the end, the meta-analysis contained nine RCTs, with a total sample size of 1,880 people. Figure 1 displays the study selection process, and Table 1 provides a summary of the fundamental traits of the included research.

**Table 1:** General information of included studies

Included literature	Publication year	Sample size	Intervention measures		EGFR mutation	Medication sequence	Outcome indicator
			Experimental group	Control group			
Mork [11]	2017	419	Osimertinib	Pemetrexed + carboplatin/cisplatin	T790M mutation	≥ second-line medication	①②③
Soria [12]	2018	556	Osimertinib	Gefitinib/erlotinib	Ex19del/L858R	First-line medication	①②③④
Nie [13]	2018	145	Osimertinib	Docetaxel + bevacizumab	T790M mutation	≥ second-line medication	①②③④
Yan Lixiang [14]	2019	64	Osimertinib	Pemetrexed + carboplatin	T790M mutation	≥ second-line medication	①②③④
Liu Dan [15]	2019	90	Osimertinib	Pemetrexed + cisplatin	T790M mutation	≥ second-line medication	①②③④
Wang Rukun [16]	2019	104	Osimertinib	Pemetrexed + cisplatin	T790M mutation	≥ second-line medication	①②③④
Dai Weijing [17]	2020	100	Osimertinib	Pemetrexed + cisplatin			
Wu Peng [18]	2024	320	Osimertinib	Gemcitabine + cisplatin	Ex19del/L858R	First-line medication	①②
Feng Yuyan [19]	2025	90	Osimertinib	Pemetrexed + cisplatin	Ex19del/L858R	First-line medication	① ②④

①ORR; ②DCR; ③ All kinds of ADRs and ADRs above level 3; ④PFS

### Evaluation of methodological quality of included studies

**Fig. 2:**
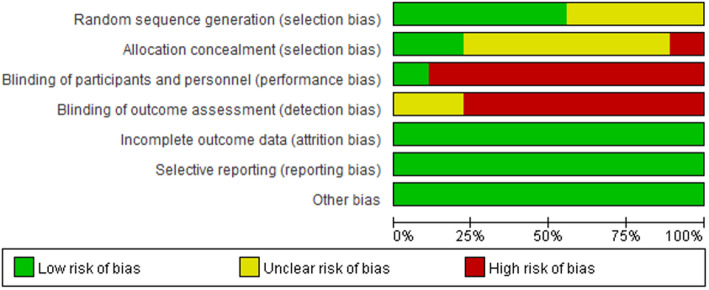
Risk Bias Graph

**Fig. 3:**
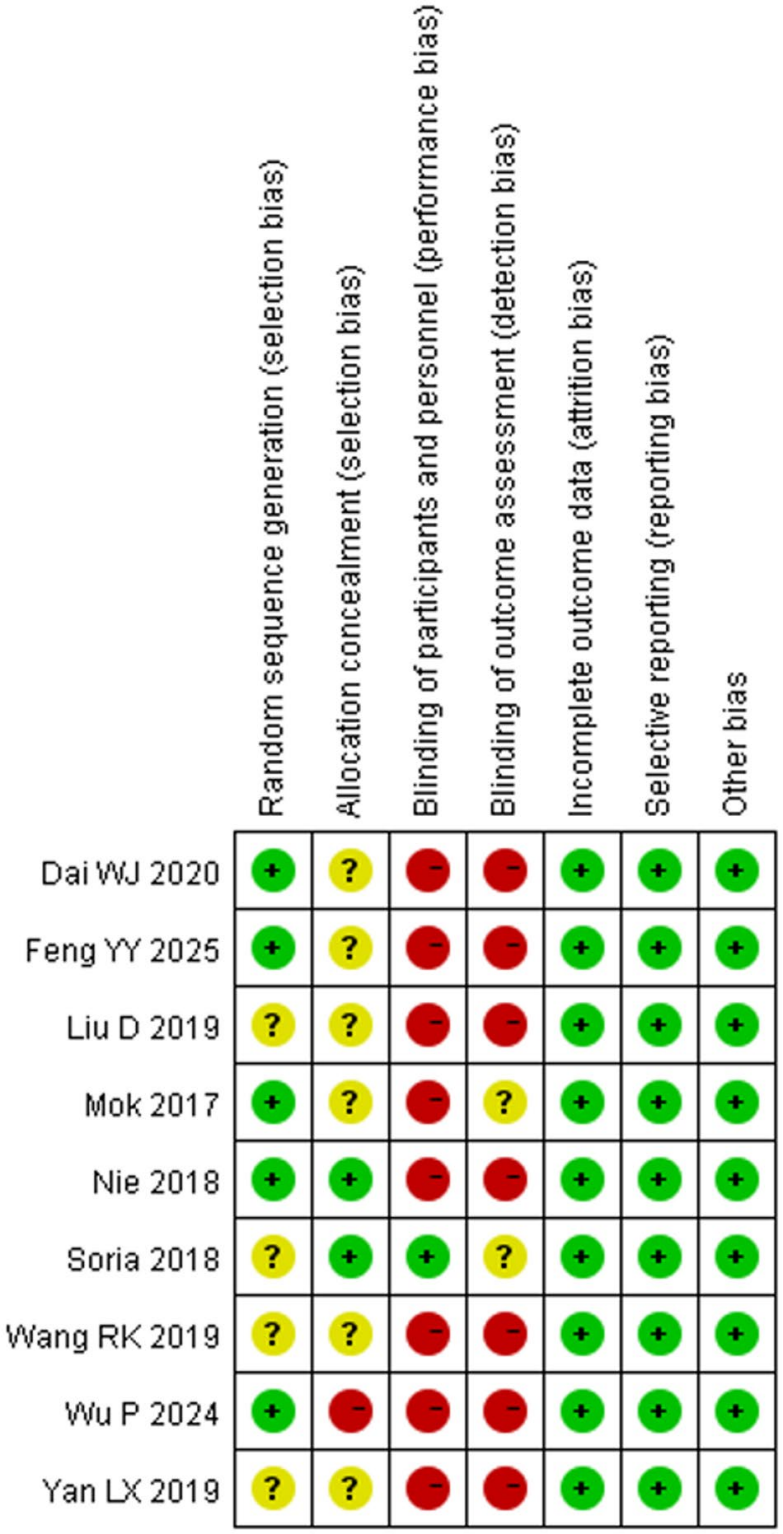
Summary of Risk of Bias Assessment

Among the 9 RCTS included in this meta-analysis, 4 literatures provided specific methods for generating random sequences, 3 literatures described the allocation hiding method, 2 literatures adopted the double-blind method to reduce implementation bias, and 2 literatures used intention-to-treat analysis to reduce the effect of incomplete data on the findings. The specific risk bias analysis is shown in Figs. 2 and 3.

### Meta-analysis results

#### Objective remission rate

**Fig. 4:**
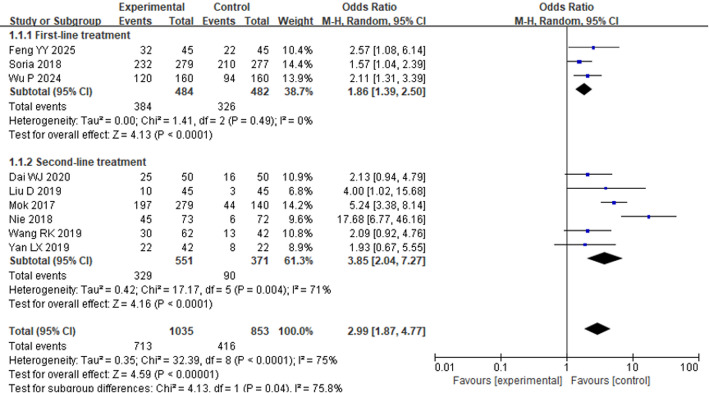
Forest plot (FP) comparing ORR between the EG and the CG

**Fig. 5:**
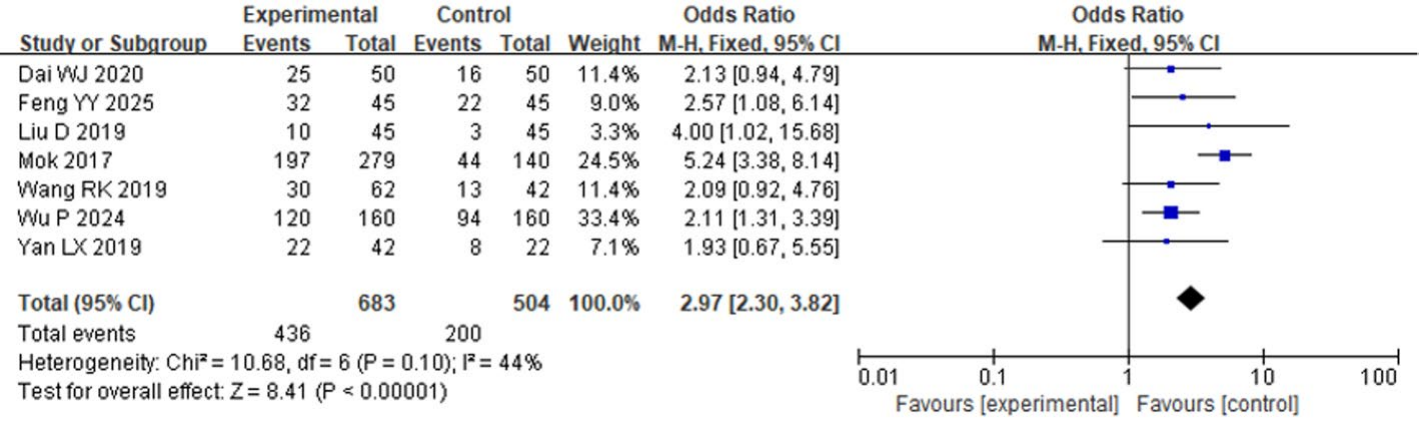
FP comparing ORR between the EG and the chemotherapy-only group

**Fig. 6:**
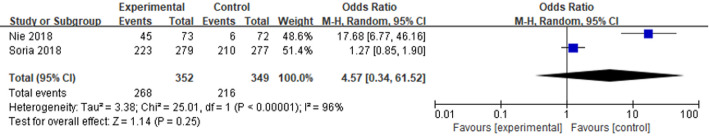
FP comparing ORR in the EG with other treatment groups

Seven studies reported the ORR of osimertinib in the treatment of individuals with advanced NSCLC. HET analysis revealed considerable HET among the included studies (*P* < 0.001, I² = 75%). Therefore, a REM was used. As shown in Fig. 4, regardless of whether osimertinib was administered across first-, second-, and beyond-line settings, the ORR in the EG was considerably higher than that in the CG [OR = 2.99, 95% CI: 1.87–4.77, *P* < 0.00001]. A subgroup analysis was carried out on the basis of the type of intervention in the CG. Among the included studies, seven compared osimertinib with chemotherapy-only regimens, while two included other therapeutic agents in the CG. In the chemotherapy-only subgroup, HET was moderate (*P* = 0.10, I² = 44.0%), and a fixed-effect model was applied. The pooled findings indicated that osimertinib significantly improved ORR in contrast to chemotherapy alone [OR = 2.97, 95% CI: 2.30–3.82, *P* < 0.00001], as shown in Fig. 5. In contrast, when osimertinib was compared to other treatment regimens (non-chemotherapy-based), substantial HET was observed (*P* < 0.00001, I² = 96%), and there was no considerably change in ORR. [OR = 4.57, 95% CI: 0.34–61.52, *P* = 0.25], as shown in Fig. 6.

#### Disease control rate

**Fig. 7:**
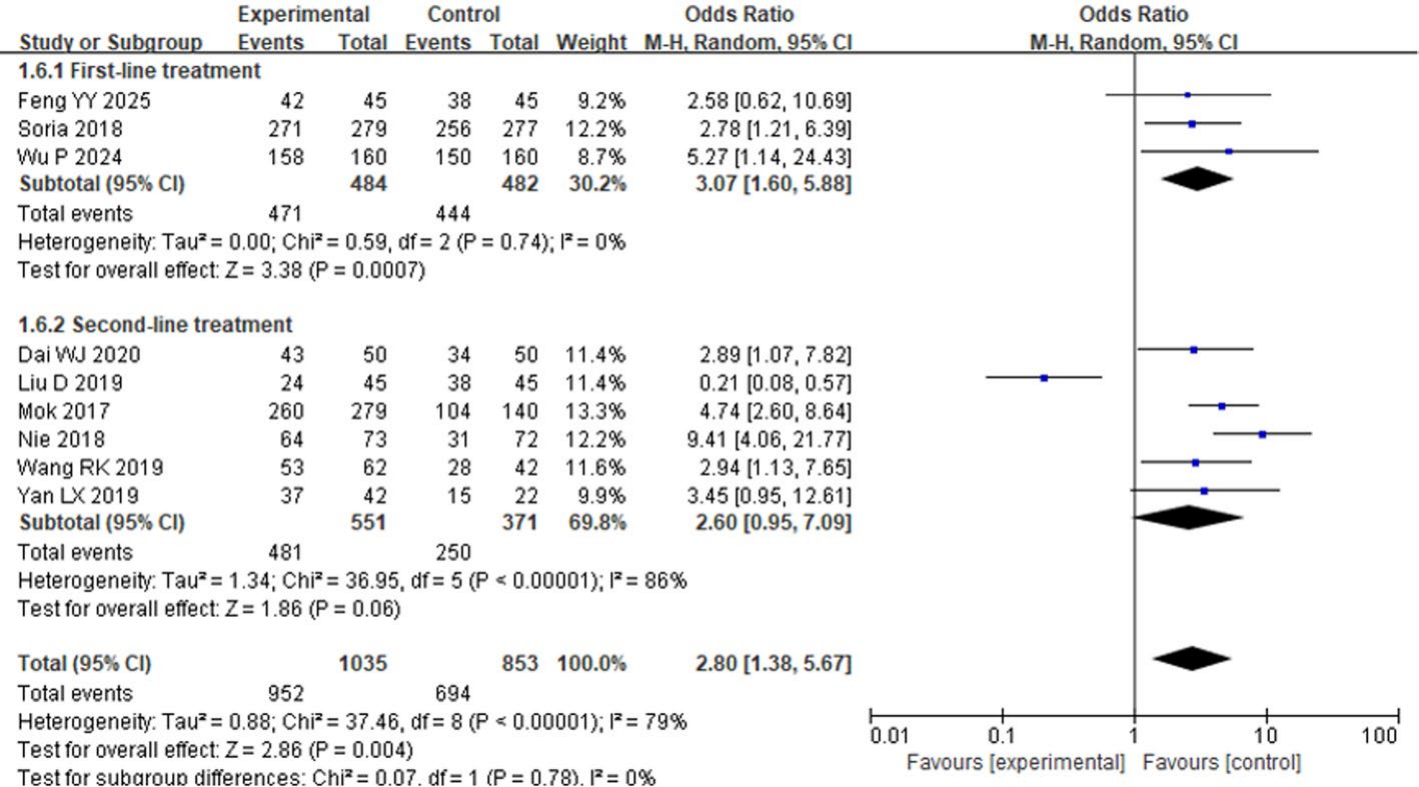
FP comparing DCR between the EG and the CG

Nine literatures reported the DCR of osimertinib in the treatment of individuals with advanced NSCLC. HET analysis was conducted on the included literatures, and no statistical HET was found (*P* < 0.00001, I^2^ = 79%). Therefore, a REM was applied for analysis. As seen in Fig. 7, the FP indicated that, regardless of whether osimertinib was used as a first-line or second-line and beyond treatment, the DCR in the EG was considerably higher than that in the CG [OR = 2.80, 95% CI: 1.38–5.67, *P* = 0.004].

#### Adverse drug reactions

**Fig. 8:**
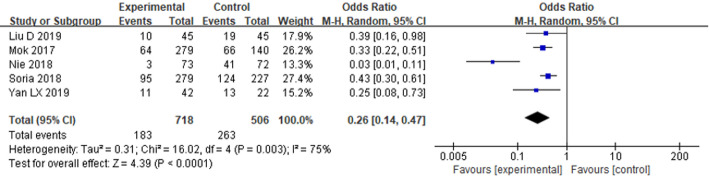
FP comparing the incidence of grade 3 and above ADR between the EG and the CG

The frequency of adverse events of grade 3 and above was included in 5 literatures, among which there were 718 cases in the EG and 506 cases in the CG. The OR values and 95% confidence intervals of the findings of the five studies were combined. The HET among the studies was large (*P* = 0.003, I^2^ = 75%), and the REMwas used for analysis. According to the FP, it can be known that compared with the CG, the incidence of grade 3 and above ADR in the EG was significantly reduced[OR = 0.26, 95%CI (0.14–0.47), *P* < 0.0001], as seen in Fig. 8.

####  Incidence of various types of ADR

Frequently reported ADRs associated with osimertinib were summarized from the included studies. The most common ADRs were gastrointestinal and dermatologic in nature, including diarrhea, rash, and paronychia. Several studies also reported a rise in interstitial lung disease cases and cardiotoxicity in the OG, with some cases resulting in fatal outcomes due to interstitial lung disease. Data on specific ADRs were extracted and subjected to meta-analysis. As shown in Table 2, patients receiving osimertinib had a significantly higher risk of developing paronychia and rash compared to the CG [OR = 3.42, 95% CI: 1.12–10.42, *P* = 0.03]. Conversely, the incidence of nausea and vomiting was considerably lower in the OG [OR = 0.27, 95% CI: 0.14–0.53, *P* < 0.001].

**Table 2:** Meta-analysis table of the incidence rates of various adverse reactions

Adverse reaction	The total number of included literatures	The number of positive events (Experimental Group/Control Group)	OR	95%CI	Heterogeneity(I%;*P*)
diarrhea	7	331/198	1.97	1.07–3.61	83;0.03
Rash	7	326/240	3.44	0.84–14.07	93;0.08
Paronychia	5	195/104	3.42	1.12–10.42	82;0.03
Nausea and vomiting	8	116/206	0.27	0.14–0.53	78;<0.001
Interstitial pneumonia	6	28/18	1.23	0.67–2.27	31;0.50
QT interval prolongation	7	54/16	2.18	0.81–5.84	46;0.08
Constipation	4	95/109	0.46	0.20–1.07	83;0.07
Anemia	6	79/99	0.51	0.21–1.23	81;0.14

#### Progress free survival

**Fig. 9:**
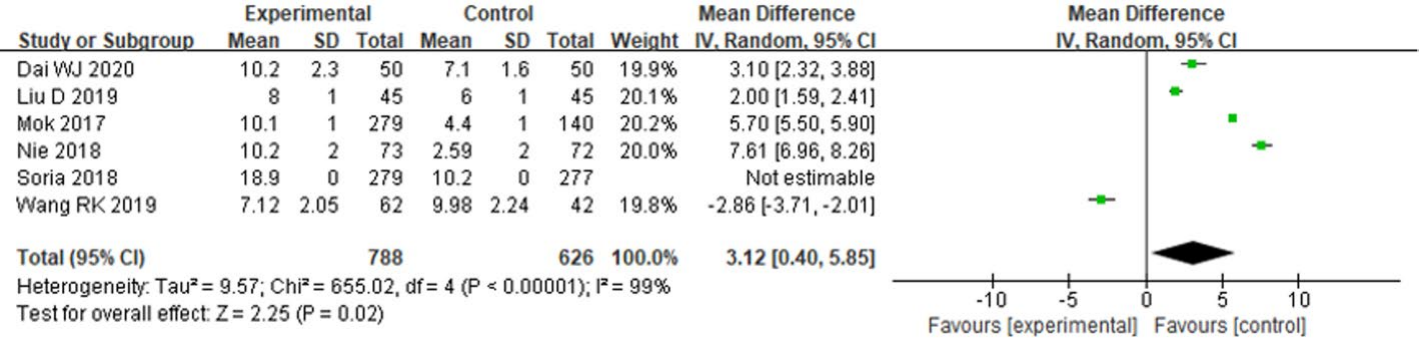
FP comparing PFS between the EG and the CG

Six literatures reported the PFS of osimertinib in the treatment of individuals with advanced NSCLC. HET analysis was conducted on the included literatures, and statistical HET was found (*P* < 0.00001, I^2=^99%). Therefore, a REM was selected for analysis. According to the FP, it can be known that the PFS of the EG was considerably prolonged compared with that of the CG [OR = 3.12, 95%CI (0.40–5.85), *P* = 0.02], as seen in Fig. 9.

## Discussion

*EGFR* gene mutation is a relatively common driver factor of NSCLC. For advanced NSCLC individuals with EGFR gene mutation and no drug resistance genes, the first-line medication is frequently advised to be EGFR-TKI. However, during the clinical treatment process, some patients will still develop drug resistance successively. Among them, acquired drug resistance caused by positive T790M mutation accounts for approximately 50% to 60%. Managing first- and second-generation EGFR-TKI resistance effectively has become a pivotal challenge in achieving precision, whole-course care for individuals with advanced NSCLC. Osimertinib represents a significant advance in this context. The agent forms an irreversible covalent bond with the cysteine residue at position 797 (C797) within the EGFR kinase domain, thereby suppressing tyrosine-kinase phosphorylation and downstream EGFR signaling. By blocking EGFR homodimerization and inhibiting activation of key proliferative pathways, osimertinib selectively induces apoptosis in EGFR-mutant tumor cells and ultimately curtails malignant proliferation [20]. At present, osimertinib has become a commonly used targeted drug in clinical practice. This study included 9 RCTs, involving a total of 1,880 patients. The meta-analysis method was employed to evaluate the security and effectiveness of osimertinib in the handling of advanced NSCLC.

In terms of therapeutic effect, this study showed that regardless of whether in first-line OR second-line and above treatments, the ORR and DCR in the OG were considerably higher than those in the CG (ORR: OR = 2.99, 95%CI:1.874.77, *P* < 0.00001; DCR: OR = 2.80, 95%CI:1.385.67, *P* = 0.004). Subgroup analysis using chemotherapy as the CG demonstrated a more pronounced therapeutic advantage of osimertinib, further reinforcing its pivotal role in the management of EGFR-mutated NSCLC. This superior efficacy may be attributed to osimertinib’s high selectivity for the EGFR T790M resistance mutation and its enhanced inhibitory activity against EGFR signaling. Additionally, osimertinib exhibits favorable blood-brain barrier penetration, enabling effective control of CNS metastases and delaying disease progression in this critical compartment. Previous studies have shown that compared with traditional chemotherapy, first-generation and second-generation EGFR-TKIs can effectively improve the prognosis of NSCLC individuals with EGFR mutations [21, 22]. The third-generation targeted drug osimertinib has stronger selectivity for EGFR mutations and a better inhibitory effect on EGFRT790M gene mutations. When EGFR-TKIs of the first and second generations become resistant, EGFRT790M mutations are prone to occur. At this time, osimertinib is a very effective treatment option. Based on this study, Cheng et al. [23] conducted research on the population in the Chinese region. Patients in the OG achieved good survival benefits. In addition, osimertinib has good penetration of the blood-brain barrier. Mok et al. conducted a subgroup analysis on 144 patients with CNS metastases [11]. Compared with patients receiving platinum-based chemotherapy, those treated with osimertinib demonstrated a significantly prolonged median survival. However, due to insufficient data in the included studies, subgroup analysis for CNS outcomes could not be performed in this meta-analysis. Both domestic and international clinical guidelines presently advise osimertinib as the standard first-line treatment for individuals with EGFR-mutated NSCLC [24], with well-established survival benefits. Nonetheless, in real-world clinical practice, treatment decisions should be made with comprehensive consideration of multiple factors, including the safety profile of osimertinib, management strategies following the development of drug resistance, and cost-effectiveness.

The findings of the meta-analysis stated that the incidence of grade 3 and above ADR in the OG was considerably lower than that in the CG (OR = 0.26, 95%CI: 0.14–0.47, *P* < 0.0001), suggesting that it has better tolerance and safety. However, it is also necessary to be vigilant about the occurrence of some specific adverse reactions, such as a significantly increased risk of skin-related events like rash and paronychia (OR = 3.42, *P* = 0.03), which is closely related to osimertinib’s inhibition of the epidermal EGFR pathway as an EGFR-TKI drug. Meanwhile, the incidence of nausea and vomiting in the OG decreased significantly (OR = 0.27, *P* < 0.001), indicating that its gastrointestinal toxicity was significantly reduced compared with chemotherapy. In contrast to the first-generation and second-generation EGFR-TKIs, it has stronger targeting and is irreversible. Therefore, the common ADRs in clinical practice are relatively mild.

Interstitial lung disease is a relatively rare ADR of osimertinib, with an incidence rate of 1% to 3%. However, it is prone to develop into severe ADR, forcing patients to discontinue the medication. Study has shown that taking osimertinib within 6 months after discontinuous PD-1 immunotherapy increases the incidence of interstitial lung disease [25]. This might be because PD-1 immunotherapy is an immunotherapy drug that can effectively inhibit tumors and it can activate the immune activity of T lymphocyte CD8^+^. Therefore, in patients with EGFR mutations, re-administration of osimertinib shortly after the failure of later-line immunotherapy is not recommended. Additionally, osimertinib is associated with a risk of cardiotoxicity, with arrhythmia—particularly QT interval prolongation—being the most notable manifestation. The incidence of QT prolongation appears to be positively correlated with both the dosage and duration of osimertinib administration. Mechanistically, this effect may be linked to osimertinib’s inhibition of the PI3K signaling pathway [26] and its potent anti-HER2 activity [27]. Although an increased incidence of rash and QT interval prolongation was observed in some studies, the meta-analysis did not reach statistical significance (rash: *P* = 0.08; QT prolongation: *P* = 0.08), and further validation is needed.

From the prognostic perspective, osimertinib significantly prolonged the PFS of patients (OR = 3.12, 95%CI: 0.40–5.85, *P* = 0.02), further demonstrating its advantage in controlling the progression of the disease. The substantial heterogeneity in PFS (I^2^ = 99%) may stem from differences in treatment lines (first-line vs. subsequent-line), EGFR mutation types (Ex19del, L858R, T790M), and demographic or geographic variation. Future studies with more granular data may help resolve this variability. The results should be comprehensively interpreted in combination with the specific clinical background.

There are several limitations to this study. For starters, there weren’t many RCTs. and the total sample size was limited, which may reduce the statistical power and particularly impact the reliability of subgroup analyses, for instance, the subgroup with alternative treatment regimens in the control arm included only two studies. Second, substantial HET was seen throughout the included studies (I² = 75%–99%), which may be attributed to variations in treatment lines (first-line vs. second-line or beyond), differences in CG interventions (e.g., chemotherapy regimens or alternative targeted therapies), and HET in EGFR mutation subtypes (such as Ex19del, L858R, and T790M). Although a REM was applied to account for this variability, residual HET may still compromise the robustness of the results. Additionally, the absence of data on key long-term prognostic indicators, such as overall survival (OS) and patient-reported quality of life, limits the ability to fully assess the comprehensive benefits of osimertinib therapy.

## Conclusion

To summarize, osimertinib, a third-generation EGFR-TKI, demonstrates remarkable clinical efficacy and a favorable safety profile in individuals with advanced NSCLC, particularly in those harboring the T790M mutation or demonstrating resistance to EGFR-TKIs of the previous generation. While most adverse reactions are manageable, certain toxicities, such as cardiotoxicity and interstitial lung disease—require enhanced clinical monitoring and management to ensure patient safety. Further real-world studies with long-term follow-up are warranted to validate these findings and assess overall survival and quality-of-life outcomes. Incorporating such evidence may refine therapeutic strategies and inform guideline recommendations.

## Data Availability

The datasets used or analyzed during the current study are available from the corresponding author on reasonable request.
